# Melatonin Implantation Improves the Reproductive Performance of Estrus-Synchronized Ewes During Seasonal Anestrus and Enhances the Antioxidant and Steroidogenic Capacities of Granulosa and Luteal Cells

**DOI:** 10.3390/antiox14070895

**Published:** 2025-07-21

**Authors:** Zengyi Duan, Menghao Liu, Junjin Li, Kexiong Liu, Qi Qi, Zhixuan Yu, Hadia Akber Samoo, Chunxin Wang, Jian Hou

**Affiliations:** State Key Laboratory of Animal Biotech Breeding, College of Biological Sciences, China Agricultural University, Yuan-Ming-Yuan West Road, Haidian District, Beijing 100193, China; zengyiduan@163.com (Z.D.); liumenghao@cau.edu.cn (M.L.); liyuhailan@126.com (J.L.); liukexiong2023@163.com (K.L.); qiqi2017@cau.edu.cn (Q.Q.); yuzhixuan@cau.edu.cn (Z.Y.); hadiaakbersamoo@stu.zafu.edu.cn (H.A.S.); wcxjlsnky@163.com (C.W.)

**Keywords:** estrus synchronization, melatonin, transcriptome, granulosa cells, luteal cells, sheep

## Abstract

Seasonal reproduction in sheep reduces reproductive efficiency. Melatonin (MT) plays a crucial role in reproductive processes. The purpose of this study was to assess the effects of a 5-day MT implant pretreatment on estrus synchronization and reproductive performance in sheep during seasonal anestrus. A total of 40 multiparous Mongolian sheep were selected and randomly divided into two groups. In the MT group (n = 20), the ewes received an MT implant for 5 days, and then, they were given a progesterone (P4)-containing vaginal sponge for 14 days with equine chorionic gonadotropin (eCG) administered (330 I.U. per ewe; I.M.) at sponge removal. Control (CON) ewes (n = 20) were similarly treated but did not receive MT implants. The results demonstrated that MT implantation significantly improved serum levels of total antioxidant capacity (T-AOC), superoxide dismutase (SOD), catalase (CAT), glutathione (GSH), and glutathione peroxidase (GSH-Px), increased post-ovulatory luteal diameter and serum P4 levels, and reduced ovarian apoptosis. Compared with the CON group, the MT group showed significantly higher pregnancy (68.23% vs. 50.59%) and lambing rates (63.53% vs. 47.06%; number of lambed ewes/number of total ewes) following cervical-timed artificial insemination. Ovarian transcriptome analysis revealed 522 differentially expressed genes (DEGs) in the MT group compared with the CON group, including 355 upregulated and 167 downregulated DEGs. In addition, MT significantly enhanced proliferation and inhibited apoptosis in cultured granulosa cells (GCs) and luteal cells (LCs) in vitro. Moreover, it enhanced the antioxidant capacity of GCs and LCs probably by activating the NRF2 signaling pathway as well as stimulating steroid hormone synthesis. In conclusion, MT implantation 5 days before applying the conventional P4-eCG protocol enhances ovine reproductive outcomes during seasonal anestrus. MT implantation has a beneficial role on the growth and function of ovarian cells. These findings offer novel evidence supporting the functional role of MT in mammalian reproduction, and would be informative for optimizing estrus synchronization in sheep.

## 1. Introduction

Reproductive activity in most sheep breeds displays seasonal variation [1]. Onset of breeding behavior is typically observed in late summer or autumn, corresponding with declining daylight hours, whilst reduced breeding activity occurs in late winter. Seasonal breeding patterns of this nature lead to fluctuations in animal productivity. To address seasonal fluctuations and sustain uninterrupted production of meat, milk, wool, and hides, out-of-season breeding strategies are required.

Estrus synchronization is usually adopted for controlling and inducing estrus and ovulation in small ruminants during both seasonal and non-seasonal breeding periods. Using estrus synchronization during anestrus increases reproductive efficiency and allows for three lambings within two years [2]. Worldwide, progesterone (P4)–equine chorionic gonadotrophin (eCG) protocols represent the most widely employed approach for estrus synchronization. Intravaginal devices containing P4 are usually in place for 12–14 days and eCG is administered at the time of device withdrawal [3,4,5,6]. However, considerable differences in reproductive outcomes are consistently observed between seasonal and non-seasonal breeding periods, and ewes often exhibit lower fertility in the non-breeding season even following induced estrus [7,8,9,10].

The reproductive seasonality of small ruminants is predominantly regulated by photoperiodic cues [11]. Melatonin (MT), secreted by the pineal gland in a light-dependent circadian manner, functions as the key hormonal messenger linking environmental photoperiod to reproductive neuroendocrine activity [12]. In short-day breeding species, including sheep and goats, extended nocturnal melatonin profiles generated by autumn photoperiods serve as the neuroendocrine trigger for seasonal reproductive activation [13]. MT is widely recognized as a crucial hormone in modulating circadian rhythms in animals [14]. The circadian rhythm controls multiple physiological processes, including thermoregulation, sleep patterns, hormonal secretion, and reproduction [14,15]. Furthermore, MT exhibits multiple biological activities, including antioxidant, anti-inflammatory, antitumor, and antiapoptotic effects [16]. In terms of reproduction, MT can promote follicular development, improve oocyte maturation, enhance embryonic development, strengthen luteal function, and support fetal development [15,16,17,18,19]. Exogenous MT is successfully used to advance the breeding season in ewes and goats by simulating the promotive influence of reduced photoperiod [20,21,22,23,24,25]. However, MT treatment must be initiated during the ‘therapeutic efficacy window,’ which is determined by the breed-specific timing of natural seasonal estrus onset [26].

MT implantation combined with the traditional P4-eCG regimen improves the reproductive outcomes of sheep during seasonal anestrus [10,27,28,29]. In those studies, however, MT implantation was mostly performed at least 26 days prior to inserting the intravaginal devices [10]. This increases the lambing interval and is not conducive to the implementation of intensive reproductive management systems, highlighting the urgency for developing more reliable hormonal synchronization strategies. Therefore, a more flexible, practical, and efficient hormonal synchronization regimen is needed in modern sheep husbandry.

This study aimed to evaluate the effects of a 5-day MT implant pretreatment prior to conventional P4–eCG synchronization protocol on reproductive performance and ovarian response in sheep during seasonal anestrus. We postulated that a 5-day MT implant pretreatment was effective for improving the reproductive outcomes in sheep during seasonal anestrus. We also aimed to understand the molecular pathways through which MT modulates ovarian cell function.

## 2. Materials and Methods

### 2.1. Ethics Approval

All animal experimental protocols were conducted in strict compliance with the Institutional Animal Care and Use Guidelines of China Agricultural University (AW20701202-3-3), following review and approval by the University’s Animal Ethics Committee.

### 2.2. Location and Animal Management

All animal experiments were conducted in the Hanshan White Cashmere Goat Breeding Farm, located at Balin Right Banner (118°15′ E, 43°12′ N), Chifeng, Inner Mongolia, China; the region is characterized by temperate continental climate with mean seasonal temperatures of 1 °C (spring) and 20.5 °C (summer). Experimental ewes (Mongolian and Hu sheep) were maintained in semi-open housing with ad libitum access to mineral supplements and water, receiving rations of corn silage–alfalfa hay mixed forage twice a day (07:00 and 17:00). 

### 2.3. Experimental Design

#### 2.3.1. Experiment 1

**Figure 1:**
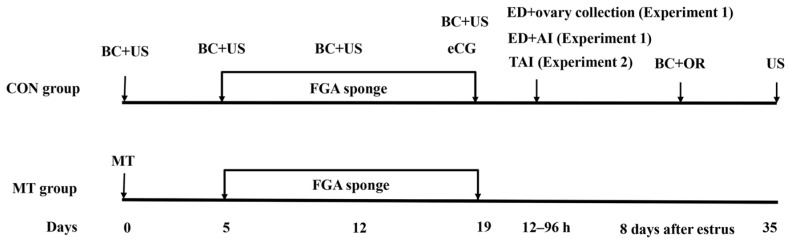
Schematic representation of the experimental workflow. CON group: FGA (45 mg) sponge for 14 days + 330 IU eCG at sponge withdrawal. MT group: Following 5 days of MT implantation, the same protocol as the CON group was applied. BC: blood collection. US: transrectal ultrasound monitoring of the ovaries. ED: estrus detection. ED + ovary collection: After estrus detection, the ovaries of ewes in estrus were collected (CON group n = 3, MT group n =4; experiment 1). AI: artificial insemination. ED + AI: Ewes in estrus were inseminated once at 12 and 24 h after estrus, respectively (experiment 1). TAI: Ewes received timed artificial insemination at 48 and 60 h post-sponge removal (experiment 2). OR: Ovulation rate was determined using transrectal ultrasound to monitor the number of corpus luteum on the ovaries 8 days after estrus (experiment 1). US: Pregnancy was diagnosed using transrectal ultrasound approximately 35 days after insemination.

In April 2021 (spring season), a total of 40 multiparous, non-lactating Mongolian ewes (aged 3–4.5 years) were stratified by initial body weight (BW) and body condition score (BCS; 1–5 scale) and randomly assigned to two experimental groups. The CON group (n = 20, BW = 49.8 ± 3.3 kg, BCS = 3.1 ± 0.2) underwent a conventional P4-eCG synchronization protocol. The ewes received intravaginal fluorogestone acetate (FGA, 45 mg)-impregnated sponges (Beijing Bevic Co., Ltd., Beijing, China) for 14 days with eCG (Ningbo Sansheng Pharmaceutical, Ningbo, China) administered (330 I.U. per ewe; I.M.) at sponge removal. In the MT group (n = 20, BW = 50.1 ± 3.9 kg, BCS = 3.2 ± 0.2), the MT-P4-eCG protocol was performed. The ewes received a single subcutaneous MT implant (18 mg, Institute of Special Animal and Plant Sciences of CAA, Changchun, China) in the left auricular base region. Five days after implantation, the sheep received the same estrus synchronization as for the CON group. The day of MT implantation was defined as Day 0. Following sponge removal, estrus detection was initiated 12 h later using teaser rams wearing a vest. Detection was performed twice daily at 12 h intervals for 4 consecutive days. Estrus was confirmed by immobility response to mounting attempts by teaser rams. The ewes exhibiting estrus were cervically artificially inseminated at both 12 and 24 h after estrus onset. Teaser rams were housed and managed separately from experimental ewes. Additionally, blood sampling and transrectal ovarian ultrasonography were performed on Day 0, 5, 12, 19, and 8 post-estrus. For transcriptome analysis, ovaries were surgically harvested from ewes during the estrus phase (CON group n = 3, MT group n = 4) (Figure 1).

#### 2.3.2. Experiment 2

Experiment 1 demonstrated better reproductive outcomes using the MT-P4-eCG protocol compared to the conventional P4-eCG treatment. To further determine the feasibility of the MT-P4-eCG protocol, in experiment 2, a field test was designed to compare the efficiency between the MT-P4-eCG and P4-eCG protocols that were used for a popular breed of Hu sheep. Hu sheep is a prevalent, intensively managed breed in China, and they also exhibit reduced reproductive performance in spring. During the spring season (April 2022), 170 multiparous Hu ewes (3–4.5 years, non-lactating) were randomly distributed to CON (n = 85, 49.3 ± 3.8 kg, BCS = 3.1 ± 0.2) or MT (n = 85; 51.2 ± 2.1 kg, BCS = 3.2 ± 0.1) groups. As timed artificial insemination (TAI) is generally used in the estrus synchronization program in Hu sheep in the farm, both the CON and MT groups received TAI at 48 and 60 h post-sponge removal. These time points (48 h and 60 h) represent empirically validated optimal intervals for Hu sheep TAI (Figure 1).

### 2.4. Artificial Insemination

Fresh semen for artificial insemination (AI) was collected via artificial vagina and evaluated for quality, with only samples exhibiting >80% progressive motility being retained for use. Semen was extended 1:1 (*v*/*v*) with a skim-milk-based diluent. For cervical AI, a sterile light-guided speculum and insemination rod (Beijing Bevic Co., Ltd., Beijing, China) were employed to deposit 0.2 mL of semen (200 × 10^6^ spermatozoa) into the cervical canal, with careful advancement to maximize intrauterine deposition. All procedures were conducted by trained technicians following standardized protocols.

### 2.5. Ultrasonography

During the treatment for estrus synchronization in experiment 1, ovarian dynamics was monitored using transrectal ultrasound (7.5 MHz, HS1600, HONDA, Tokyo, Japan), and the number and relative position of the diameters of all ≥2 mm follicles on the ovaries, as well as the number and diameter of the corpus luteum (CL), were recorded (on Day 0, 5, 12, 19, and 8 post-estrus; experiment 1). Follicles were classified into three types: small follicles (≥2 to 3 mm), medium follicles (>3 to <5 mm), and large follicles (≥5 mm) [30]. Pregnancy was diagnosed using transrectal ultrasound approximately 35 days after insemination.

### 2.6. Serum Collection and Analysis

Blood was collected from the jugular vein of ewes (on Day 0, 5, 12, 19, and 8 post-estrus; experiment 1), followed by centrifugation at 1400 g for 15 min, and the serum was stored at −20 °C. MT, P4, and insulin-like growth factor 1 (IGF-1) levels in serum were determined following the standardized procedures provided with the commercial assay kits (Beijing North Institute of Biological Technology, Beijing, China). The levels of total antioxidant capacity (T-AOC), superoxide dismutase (SOD), catalase (CAT), glutathione (GSH), glutathione peroxidase (GSH-Px), and malondialdehyde (MDA) in serum were quantified using standardized commercial assay kits (Nanjing Jiancheng Bioengineering Institute, Nanjing, China).

### 2.7. Ovarian Sample Collection

After estrus detection (ewes in experiment 1), the ovaries where the largest follicles were located in the sheep in estrus were excised by surgical method (CON group n = 3, MT group n = 4; experiment 1), and the ovaries were washed with cold saline to remove the blood on the surface, after which the ovaries were divided into two parts, one of which was fixed and preserved in 4% paraformaldehyde (Beyotime, Shanghai, China) and the other was frozen and preserved in liquid nitrogen for subsequent transcriptome analysis.

### 2.8. Ovarian Transcriptome Analysis

To elucidate the underlying molecular mechanisms by which MT enhances reproductive performance, transcriptome sequencing analysis was performed on ovary samples collected as described above. The library construction and sequencing procedures were performed following the company’s standard protocols (Berry Genomics, Beijing, China). Briefly, the library preparation workflow included RNA extraction, RNA quality assessment, library construction, library quality control, and sequencing. After completing the quality evaluation of sequencing data, reference genome alignment, and gene expression quantification, differential gene expression analysis across samples was performed using edge R software (version 3.3.3). The screening criteria were set as padj < 0.05 & |log2 FoldChange| > 1. The enrichment analysis of Gene Ontology (GO) terms for differentially expressed genes (DEGs) was carried out utilizing the topGO software package (https://bioconductor.org/packages/release/bioc/html/topGO.html, accessed on 9 September 2021), whereas the enrichment analysis of KEGG pathways was performed with the aid of the KOBAS software tool (version 3.0).

### 2.9. Granulosa Cell and Luteal Cell Collection

Ovaries were harvested from adult ewes that had been freshly slaughtered at a local abattoir (Hongfa Co., Ltd., Chifeng, Inner Mongolia, China). These ovaries were promptly transported to the laboratory within 2 h. Upon arrival, the ovaries were thoroughly rinsed with physiological saline. For isolation of granulosa cells (GCs), surface follicles (2–5 mm) were aspirated using a 10 mL syringe with 2 mL Dulbecco’s modified eagle medium (DMEM)/F12 medium (Gibco, Waltham, MA, USA), and the collected fluid was centrifuged at 1000× *g* for 5 min, after which the pellet was resuspended in DMEM/F12 supplemented with 10% fetal bovine serum (FBS) (HyClone, Logan, UT, USA) and 1% penicillin–streptomycin (Gibco) and cultured in 6-well plates (Corning, New York, NY, USA) at 37 °C under 5% CO_2_. For isolation of luteal cells (LCs), the CL was decapsulated, minced into small pieces, and evenly plated in a 60 mm culture dish (Corning) with a small volume of DMEM/F12 containing 10% FBS and 1% penicillin–streptomycin, followed by incubation (37 °C, 5% CO_2_) for 4–6 h to enable adherence before adding complete medium, with subsequent medium changes every 48 h until confluence.

### 2.10. Cell Counting Kit-8 (CCK-8) Assay

Cell viability was assessed utilizing the enhanced Cell Counting Kit-8 (CCK-8) assay (Beyotime). The cells were initially seeded into 96-well plates (Corning) and cultured until they achieved 70–80% of confluency. Subsequently, the cells underwent serum deprivation for a duration of 12 h, after which they were exposed to various concentrations of MT (10^−7^, 10^−8^, and 10^−9^ M, Sigma-Aldrich, MO, USA) in a serum-free medium for periods of 24, 36, and 48 h. Post-MT treatment, 10 μL of CCK-8 solution was introduced into each well, and the plates were incubated for an additional 2 h in a cell culture incubator. Finally, the absorbance was quantified at a wavelength of 450 nm in Microplate Reader (Thermo Fisher Scientific, Waltham, MA, USA).

### 2.11. 5-Ethynyl-2′-Deoxyuridine (EdU) Assay

The proliferation of GCs and LCs was evaluated utilizing an EdU assay kit, adhering strictly to the manufacturer’s instructions (Beyotime). In detail, cells were incubated with a prewarmed (37 °C) 2×EdU working solution for 2 h. Afterwards, they were fixed with 4% paraformaldehyde at room temperature for 15 min. Following fixation, cells were permeabilized by treatment with PBS containing 0.3% Triton X-100 for 15 min. Subsequently, the click reaction mixture was added, and the cells were incubated in the dark for 30 min. Nuclei were then counterstained with Hoechst 33342 for 10 min. Finally, the cells were rinsed three times with PBS and examined under a fluorescence microscope (Olympus, BX51, Tokyo, Japan).

### 2.12. Terminal Transferase dUTP Nick-End Labeling (TUNEL) Assay

Apoptosis in ovarian tissue, GCs, and LCs was detected using a one-step TUNEL apoptosis detection kit (Beyotime), following the manufacturer’s guidelines. For ovarian tissue sections, the process began with dewaxing and rehydration, after which the sections were treated with proteinase K at 37 °C for 30 min, followed by three washes with PBS. The TUNEL reaction mixture was then applied to the samples and incubated at 37 °C in the dark for 60 min. After three PBS washes, 4′,6-diamidino-2-phenylindole (DAPI) was added and incubated for 10 min. Finally, the sections were washed three times with PBS and examined under a fluorescence microscope (Olympus). For GCs and LCs, the cells were initially fixed with 4% paraformaldehyde for 30 min and then washed once with PBS. Subsequently, the cells were incubated with PBS containing 0.3% Triton X-100 at room temperature for 5 min. Following two PBS washes, the TUNEL reaction mixture was added to the samples, and the samples were then incubated at 37 °C in the dark for 60 min. The subsequent procedures were the same as those described for the TUNEL assay of the ovarian tissue sections.

### 2.13. Reactive Oxygen Species (ROS) Assay

ROS levels were quantified utilizing a ROS detection assay kit (Beyotime), strictly adhering to the manufacturer’s prescribed protocols. Specifically, DCFH-DA was diluted at a ratio of 1:1000 in DMEM/F12 culture medium and subsequently added to the cells. The cells were then incubated at 37 °C in the dark for 20 min. Post-incubation, the cells were washed three times with DMEM/F12 culture medium, and ROS production was visualized and assessed under a fluorescence microscope (Olympus).

### 2.14. Mitochondrial Membrane Potential (MMP) Assay

An enhanced MMP detection assay kit (Beyotime) was utilized to assess MMP levels in the cells. In brief, the original culture medium was carefully removed by aspiration, and an equivalent volume of JC-1 staining working solution mixed with DMEM/F12 culture medium was added to the cells. These cells were subsequently incubated at 37 °C in the dark for 20 min. Following incubation, the cells were washed twice with PBS and then examined under a fluorescence microscope (Olympus).

### 2.15. Quantitative Real-Time PCR (qRT-PCR)

Total RNA was extracted from both cells and ovarian tissues utilizing Trizol reagent (Tiangen Biotech, Beijing, China). Subsequently, cDNA synthesis was carried out employing the FastKing gDNA Dispelling RT SuperMix kit (Tiangen Biotech). Quantitative real-time PCR (qRT-PCR) was then performed using the synthesized cDNA as a template, in strict accordance with the guidelines provided in the manufacturer’s manual for the TransStart^®^ Tip Green qPCR SuperMix (+Dye II) kit (TransGen Biotech, Beijing, China). The *ACTB* gene served as the internal reference control for normalization. Data analysis was executed using the 2^−ΔΔCT^ method. The primer sequences employed in this study are detailed in Supplementary Table S1.

### 2.16. Western Blot Analysis

Cells were lysed with RIPA buffer (Beyotime) containing phosphatase inhibitor (Beyotime) and phenylmethanesulfonyl fluoride (PMSF) (Beyotime) protease inhibitor to extract proteins. Protein concentrations were measured using the BCA kit (Beyotime). After SDS-PAGE electrophoresis, the proteins were transferred onto a PVDF membrane (Millipore, Burlington, MA, USA). Subsequently, the membrane was blocked with 5% skim milk (Beyotime) at room temperature for 2 h. After that, it was incubated with the primary antibody at 4 °C for 12 h. After incubation, the membrane was washed three times with TBST (Solarbio, Beijing, China) and was further incubated with the secondary antibody at room temperature for 1.5 h. Following this, the membrane was washed another three times with TBST. ECL substrate solutions A and B (Beyotime) were mixed in a 1:1 (*v*/*v*) ratio and applied evenly onto the membrane. Chemiluminescence signals were detected using a fully automated chemiluminescence imaging system (Tanon, Shanghai, China). The detailed information on the antibodies used in this study is provided in Supplementary Table S2.

### 2.17. Statistical Analyses

All statistical analyses were conducted utilizing SPSS software (Version 26.0; IBM, Chicago, IL, USA). For the data pertaining to follicular characteristics, serum hormonal levels, and antioxidant indices, repeated measures analysis of variance was employed. Subsequent post hoc pairwise comparisons were performed using the Bonferroni test to identify specific differences among groups. The data on cell viability were analyzed via one-way ANOVA, and Duncan’s multiple range test was then carried out for multiple comparisons among the groups. When comparing estrus, pregnancy, and lambing rates, the Fisher exact test or Chi-square test was selected based on the sample size. The remaining datasets were analyzed using Student’s *t*-test. Continuous variables are reported as the mean ± standard error of the mean (SEM), while categorical variables are presented as percentages. A *p* value of less than 0.05 was considered statistically significant throughout the study.

## 3. Results

### 3.1. Effects of Melatonin on Hormonal Levels, Antioxidant Indices, and Ovarian Dynamics

**Figure 2:**
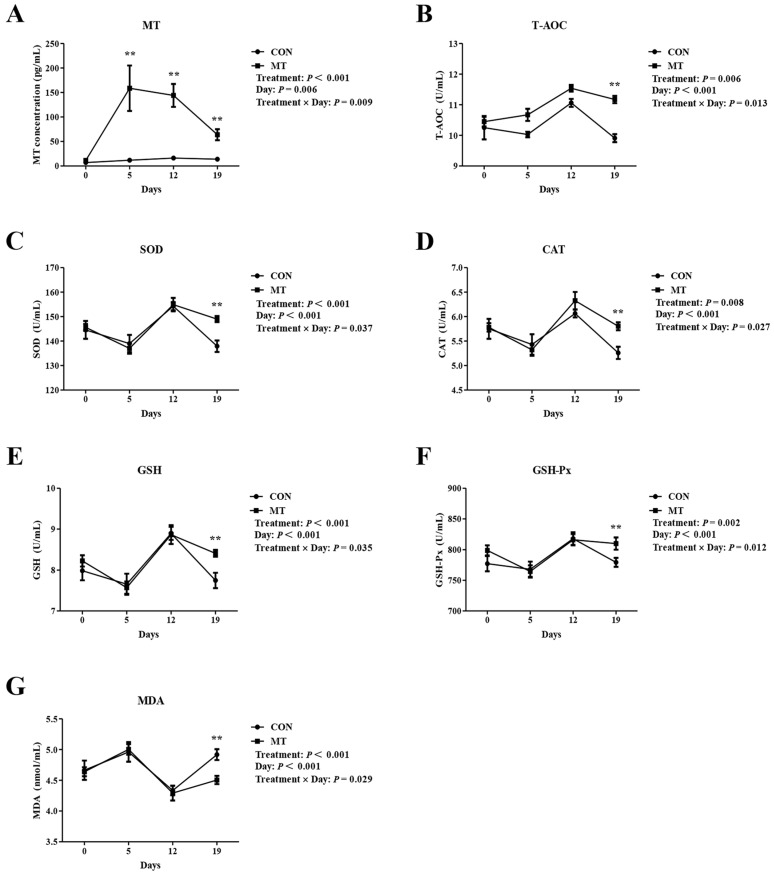
Effects of melatonin on serum antioxidant indices. CON group: FGA (45 mg) sponge for 14 days + 330 IU eCG at sponge withdrawal. MT group: Following 5 days of MT implantation, the same protocol as the CON group was applied. Days 0, 5, 12, and 19 represent MT implantation, sponge insertion, 7 days post-sponge insertion, and sponge withdrawal, respectively. (**A**) Serum MT concentration. (**B**–**G**) Serum T-AOC, SOD, CAT, GSH, GSH-Px, and MDA levels. Results are expressed as Mean ± SEM (n = 10). ** *p* < 0.01.

The effects of MT implantation on serum concentrations of MT were examined. As shown in Figure 2A, a marked increase in serum MT levels was observed in the MT group on Day 5 and Day 12. Although it decreased on Day 19, the MT level in the MT group was still significantly higher than that in the CON group (*p* < 0.01). The blood samples were also used to detect serum antioxidant related indicators. The results are shown in Figure 2B–G. On Day 0, Day 5 and Day 12, no statistically significant variations were observed in serum levels of T-AOC, SOD, CAT, GSH, and GSH-Px, or MDA between the MT and CON groups (*p* > 0.05). However, on Day 19, the levels of T-AOC, SOD, CAT, GSH, and GSH-Px in the MT group were significantly higher (*p* < 0.01), but the MDA content was significantly lower (*p* < 0.01), than those in the CON group. These findings suggested that an MT implantation had the potential to enhance the serum antioxidant capacity of ewes.

**Figure 3:**
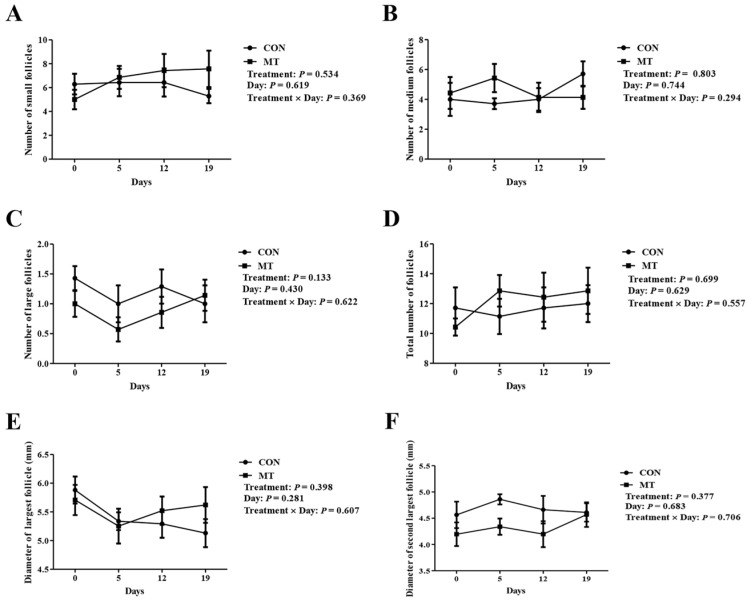
Effects of melatonin on follicular dynamics. CON group: FGA (45 mg) sponge for 14 days + 330 IU eCG at sponge withdrawal. MT group: Following 5 days of MT implantation, the same protocol as the CON group was applied. Days 0, 5, 12, and 19 represent MT implantation, sponge insertion, 7 days post-sponge insertion, and sponge withdrawal, respectively. (**A**) Number of small follicles (≥2 to 3 mm). (**B**) Number of medium follicles (>3 to <5 mm). (**C**) Number of large follicles (≥5 mm). (**D**) Total number of follicles. (**E**) Diameter of the largest follicle. (**F**) Diameter of the second largest follicle. Results are expressed as Mean ± SEM (n = 7). There were no significant differences between the experimental groups (*p* > 0.05).

To assess the impact of MT implantation on follicle growth, the number and diameter of follicles on the ovaries were detected and counted at several key time points. As depicted in Figure 3A–F, MT treatment did not alter follicle population dynamics, including counts of small, medium, or large follicles, total number of follicles, and the diameters of the two largest follicles. These results indicated that MT treatment had no significant effects on the number of developing follicles (number of follicles above 2 mm) and follicle diameter (maximum and second largest follicle diameter).

### 3.2. Effects of Melatonin on Ovarian Apoptosis and Corpus Luteum Development

**Figure 4:**
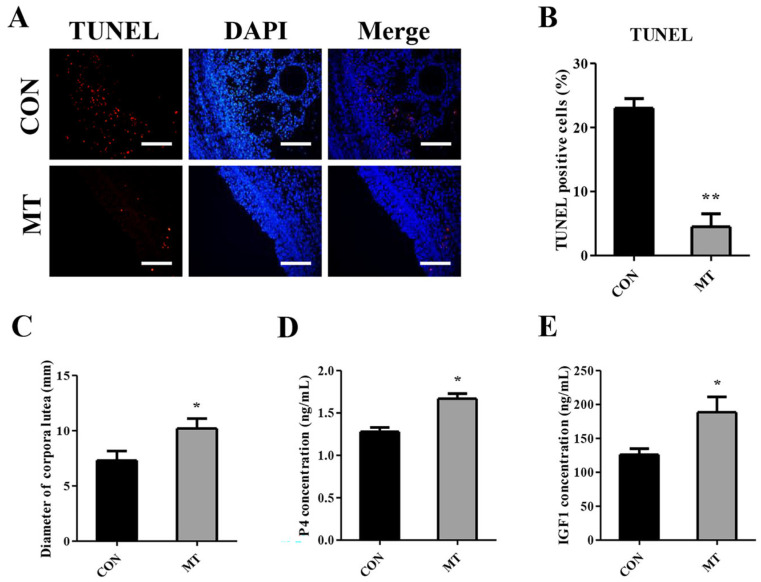
Effects of melatonin on the anti-apoptosis of ovaries and development of corpus luteum. CON group: FGA (45 mg) sponge for 14 days + 330 IU eCG at sponge withdrawal. MT group: Following 5 days of MT implantation, the same protocol as the CON group was applied. (**A**,**B**) Representative images and quantified results of TUNEL (n = 3). Scale bars = 50 μm. (**C**) Diameter of CL 8 days after estrus (n = 10). (**D**) The serum P4 concentration 8 days after estrus (n = 10). (**E**) The serum IGF-1 concentration 8 days after estrus (n = 10). Results are expressed as Mean ± SEM. * *p* < 0.05; ** *p* < 0.01.

The TUNEL assay was performed on paraffin sections of ovaries to examine ovarian cell apoptosis. The MT group exhibited significantly fewer TUNEL-positive cells compared to the CON group (*p* < 0.01), suggesting reduced apoptotic activity (Figure 4A,B).

To assess the impact of MT implantation on CL development subsequent to ovulation, we measured the diameter of the CL on the ovaries 8 days post-estrus. The results demonstrated that MT implantation led to a significant augmentation in the diameter of the CL formed after ovulation (Figure 4C). When the concentration of P4 in serum was detected 8 days after estrus, the results showed that MT implantation significantly increased serum P4 concentration (Figure 4D). MT implantation also markedly increased the serum IGF-1 concentration (Figure 4E). These results suggest that MT treatment can promote CL development and function.

### 3.3. Effects of Melatonin on Reproductive Performance

**Figure 5:**
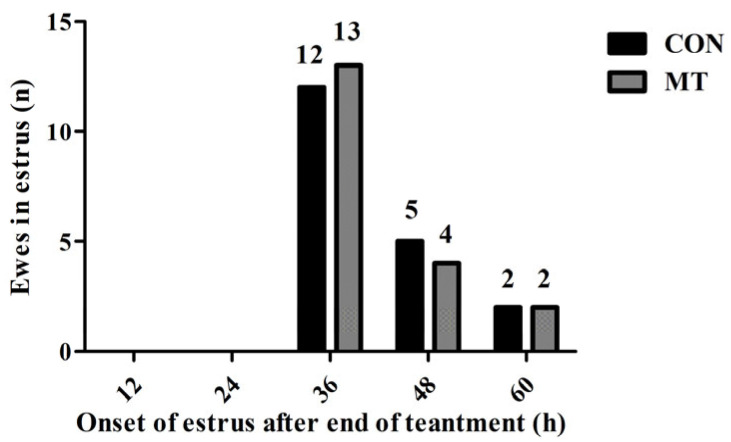
Effects of melatonin on estrus synchrony. CON group: FGA (45 mg) sponge for 14 days + 330 IU eCG at sponge withdrawal. MT group: Following 5 days of MT implantation, the same protocol as the CON group was applied.

To examine the effects of MT implantation on estrus synchrony, the distribution of estrus onset time was counted. The estrus onset of both groups was mainly synchronized at 36 h after sponge withdrawal, followed by 48 h after sponge withdrawal (Figure 5). Implantation of MT had no significant effects on estrus synchrony.

The effects of MT on reproductive performance were then evaluated. As shown in Table 1, no statistically significant difference was observed in the estrus and ovulation rates when comparing the two groups (*p* > 0.05). The MT group exhibited numerically greater pregnancy rates (86.00% vs. 56.25%) and lambing rates (86.00% vs. 56.25%) in comparison to the CON group; however, these differences did not reach statistical significance. (*p* > 0.05). There was no statistically significant variation in litter size between the two groups (*p* > 0.05). Based on the above results, while MT implantation did not result in increased estrus and ovulation, it numerically increased pregnancy and lambing rates, although the differences were not significant.

### 3.4. Effect of MT Implantation on Reproductive Performance of Ewes in a Field Test

A field test with large sample of ewes was designed to further examine the effects of MT implantation on pregnancy and lambing rates. As shown in Table 2, pregnancy and lambing rates were significantly higher in the MT group than in the CON group (*p* < 0.05). This result support the beneficial role of MT on reproduction of ewes during seasonal anestrus.

### 3.5. Effects of Melatonin on the Ovarian Transcriptome

The above experimental results showed that the implantation of MT improved reproductive performance. To explore the molecular mechanism by which MT improves reproductive performance, transcriptome sequencing analysis was performed on the ovaries sampled from ewes during estrus.

**Figure 6:**
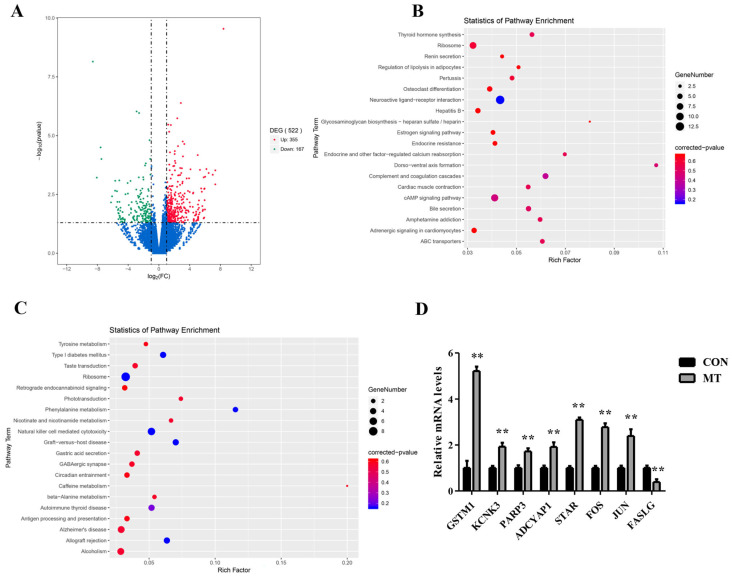
Effects of melatonin on the transcriptome of ovaries. CON group: FGA (45 mg) sponge for 14 days + 330 IU eCG at sponge withdrawal. MT group: Following 5 days of MT implantation, the same protocol as the CON group was applied. (**A**) Volcano plots of DEGs in the ovaries in the MT group vs. CON group. Upregulated genes are shown in red (*p* < 0.05), downregulated genes are shown in green (*p* < 0.05), and genes with no significant difference in expression are indicated in blue (*p* > 0.05). (**B**,**C**) KEGG enrichment analysis of upregulated and downregulated DEGs. The 20 most significantly enriched pathways are shown. (**D**) The 8 DEGs (*GSTM1*, *KCNK3*, *PARP3*, *ADCYAP1*, *STAR*, *FOS*, *JUN*, and *FASLG*) were validated by qRT-PCR. ** *p* < 0.01.

In the comparison between the MT group and the CON group, 522 DEGs were identified, comprising 355 upregulated and 167 downregulated genes in the MT group, suggesting that the implantation of MT potentially affected ovarian gene expression (Figure 6A). GO analysis identified significant enrichment of upregulated DEGs in 45 biological processes (BP), 30 cellular component (CC), and 50 molecular function (MF) terms (Figure S1A–C). Among these, several BP terms are associated with oxidative stress, DNA damage repair, and apoptosis, such as ‘positive regulation of double-strand break repair via nonhomologous end joining,’ ‘positive regulation of double-strand break repair,’ ‘regulation of double-strand break repair,’ and ‘positive regulation of DNA repair. The results suggest that MT implantation significantly attenuated DNA damage in ovarian cells. The downregulated DEGs were significantly enriched in a total of 11 BP terms (Figure S1D), 21 CC terms (Figure S1E), and 7 MF terms (Figure S1F).

Functional annotation of upregulated DEGs through KEGG pathway analysis identified 21 significantly overrepresented signaling pathways (Figure 6B). Notably, the cAMP signaling pathway and estrogen signaling pathway are implicated in steroid hormone synthesis, as well as the regulation of cell death and survival. The downregulated DEGs were mainly enriched in 20 signaling pathways (Figure 6C). In particular, the natural killer cell-mediated cytotoxicity signaling pathway is closely associated with apoptosis.

To confirm the reliability of the transcriptome sequencing results, eight DEGs (*GSTM1*, *KCNK3*, *PARP3*, *ADCYAP1*, *STAR*, *FOS*, *JUN*, and *FASLG*) related to follicular development, apoptosis, oxidative stress, DNA damage repair, and steroid hormone synthesis were randomly chosen for validation by qRT-PCR. As shown in Figure 6D, the trends of gene expression were consistent with the transcriptome sequencing data, which supported the reliability of the transcriptome data.

### 3.6. Melatonin Stimulates Proliferation While Suppressing Apoptosis in Cultured Granulosa and Luteal Cells

**Figure 7:**
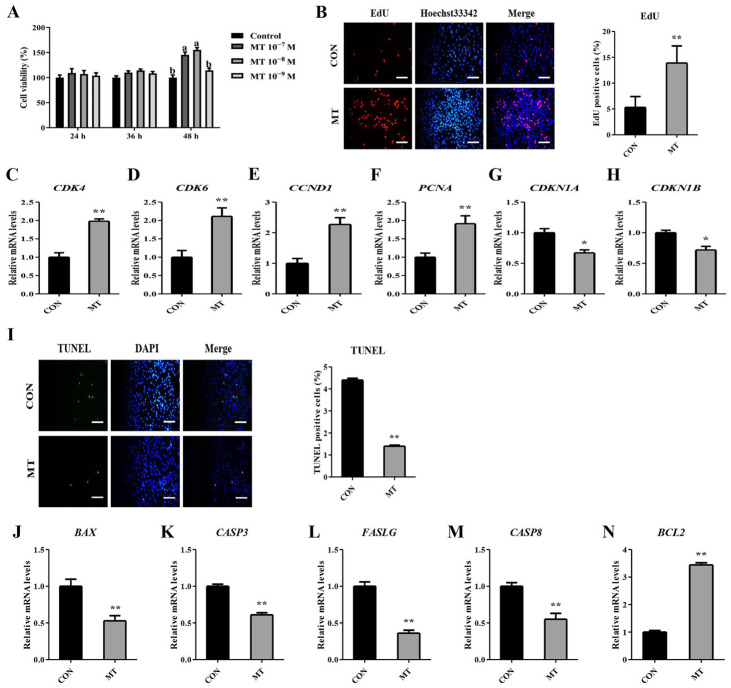
Effects of melatonin on cell proliferation and apoptosis of granulosa cells. CON group: Untreated. MT group: Cells were treated with 10^−8^ M MT in serum-free medium for 48 h. (**A**) The cell viability was determined by the CCK8 assay. (**B**) Representative images and quantified results of EdU. Scale bars = 50 μm. (**C**–**H**) The mRNA expression levels of *CDK4*, *CDK6*, *CCND1*, *PCNA*, *CDKN1A*, and *CDKN1B* were determined by qRT-PCR. (**I**) Representative images and quantified results of TUNEL. Scale bars = 50 μm. (**J**–**N**) The mRNA expression levels of *BAX*, *CASP3*, *FASLG*, *CASP8*, and *BCL2* were determined by qRT-PCR. Results are expressed as Mean ± SEM (n = 3). Within each time point, groups with different lowercase letters indicate statistically significant differences (*p* < 0.05); * *p* < 0.05; ** *p* < 0.01.

To further explore the function of MT on ovarian cells, an MT treatment was applied on GSs and LCs cultured in vitro. The GCs were treated with 10^−7^, 10^−8^, and 10^−9^ M MT for 24, 36, and 48 h, respectively, and the cell viability was detected by the CCK-8 kit. As delineated in Figure 7A, there was no significant difference in cell viability between the groups at 24 and 36 h after MT treatment. At 48 h, cells treated with 10^−7^ and 10^−8^ M MT exhibited significantly enhanced viability compared to the CON group. The cell viability of the 10^−8^ M MT group was numerically higher than that of the 10^−7^ M MT group. Therefore, subsequent experimental procedures employed a 48 h treatment with 10^−8^ M MT. EdU detection of the proliferation of GCs and LCs showed that the number of EdU-positive cells was significantly higher in the MT group than in the CON group (Figure 7B, Figure S2A). qRT-PCR analysis revealed that MT treatment significantly upregulated cell cycle activators (*CDK4*, *CDK6*, *CCND1*, and *PCNA*) while concurrently suppressing cell cycle inhibitors (*CDKN1A* and *CDKN1B*) in GCs and LCs (Figure 7C–H, Figure S2B–G). Collectively, these data indicated that MT enhanced the proliferative capacity of both GCs and LCs under in vitro conditions.

In addition, there was a marked decline in the apoptosis rate of GCs and LCs treated with MT when detected by the TUNEL method (Figure 7I, Figure S2H). MT markedly reduced the mRNA abundance of pro-apoptotic genes *BAX*, *CASP3*, *FASLG*, and *CASP8*, and notably increased the mRNA abundance of anti-apoptotic gene *BCL2* in GCs and LCs (Figure 7J–N, Figure S2I–M). Taken together, these results suggested that MT could inhibit apoptosis of GCs and LCs in vitro.

### 3.7. Melatonin Enhances the Antioxidant Capacity in Cultured Granulosa and Luteal Cells

**Figure 8:**
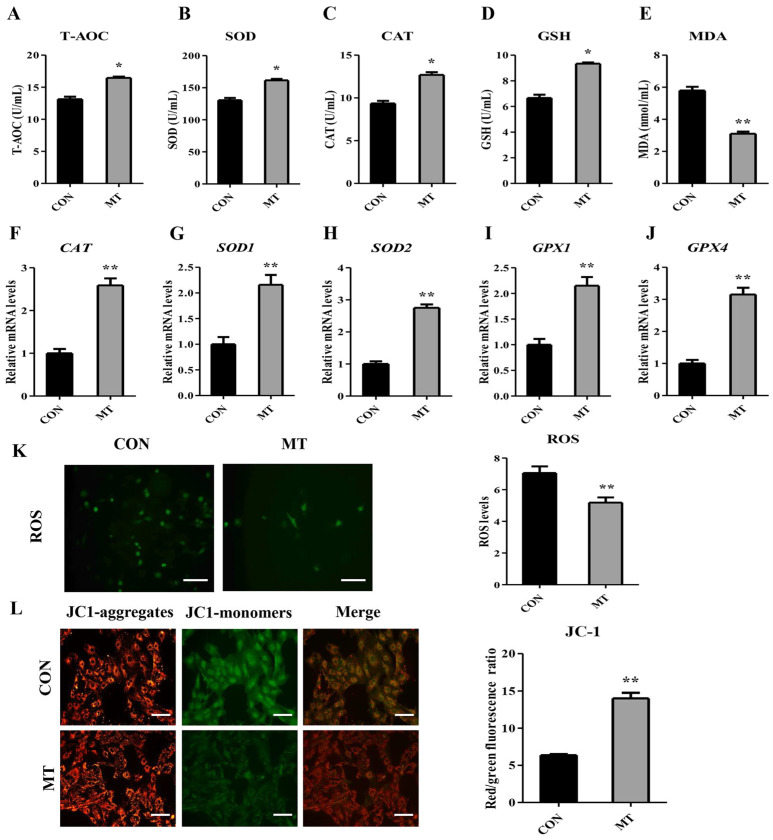
Effects of melatonin on the antioxidant capacity of granulosa cells. CON group: Untreated. MT group: Cells were treated with 10^−8^ M MT in serum-free medium for 48 h. (**A**–**E**) Levels of T-AOC, SOD, CAT, GSH, and MDA in the culture medium (n = 6). (**F**–**J**) The mRNA expression levels of *CAT*, *SOD1*, *SOD2*, *GPX1*, and *GPX4* were determined by qRT-PCR (n = 3). (**K**) Representative images and quantified results of ROS (n = 3). Scale bars = 50 μm. (**L**) Representative images and quantified results of MMP (n = 3). Scale bars = 50 μm. Results are expressed as Mean ± SEM. * *p* < 0.05; ** *p* < 0.01.

The supernatants of GC- and LC-cultured medium were collected to detect the antioxidant index. MT markedly enhanced the activities of T-AOC, SOD, CAT, and GSH content, but decreased the levels of MDA compared to the CON group (Figure 8A–E, Figure S3A–E). MT also significantly elevated mRNA abundance of *CAT*, *SOD1*, *SOD2*, *GPX1*, and *GPX4* in GCs and LCs (Figure 8F–J, Figure S3F–J).

Next, ROS and MMP were determined in GCs and LCs. The results showed that MT significantly decreased the ROS level in GCs and LCs (Figure 8K, Figure S3K), and remarkably increased the MMP in the cells (Figure 8L, Figure S3L).

**Figure 9:**
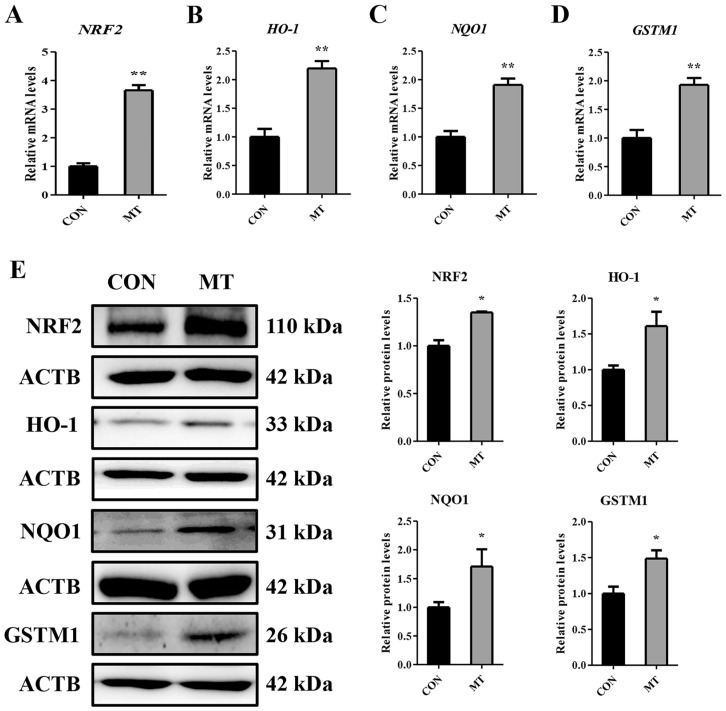
Effects of melatonin on NRF2 signaling pathway in granulosa cells. CON group: Untreated. MT group: Cells were treated with 10^−8^ M MT in serum-free medium for 48 h. (**A**–**D**) The mRNA expression levels of *NRF2*, *HO-1*, *NQO1*, and *GSTM1* were determined by qRT-PCR. (**E**) The protein expression levels of NRF2, HO-1, NQO1, and GSTM1 were determined by the Western blot analysis. Results are expressed as Mean ± SEM (n = 3). * *p* < 0.05; ** *p* < 0.01.

Transcriptomic analysis revealed significantly elevated expression of the glutathione S-transferase M1 (GSTM1) gene in the MT group compared to the CON group. Nuclear factor erythroid 2-related factor 2 (NRF2) serves as a master transcriptional regulator of GSTM1, directly interacting with antioxidant response elements (AREs) within the promoter regions of cytoprotective genes to orchestrate their transcriptional activation [31,32,33]. The NRF2-mediated antioxidant response pathway constitutes a central cellular defense mechanism against oxidative damage [34]. To further confirm the influence of MT on the NRF2 signaling pathway in GCs and LCs, qRT-PCR and the Western blot analysis were performed on the genes related to the NRF2 signaling pathway. As shown in Figure 9A–E and Figure S4A–E, compared to the CON group, MT markedly upregulated both mRNA transcripts and protein products of NRF2, HO-1, NQO1 and GSTM1 in GCs and LCs. These findings indicated that MT could activate the NRF2 signaling axis in GCs and LCs.

### 3.8. Melatonin Enhances Steroidogenic Capacity in Cultured Granulosa and Luteal Cells

**Figure 10:**
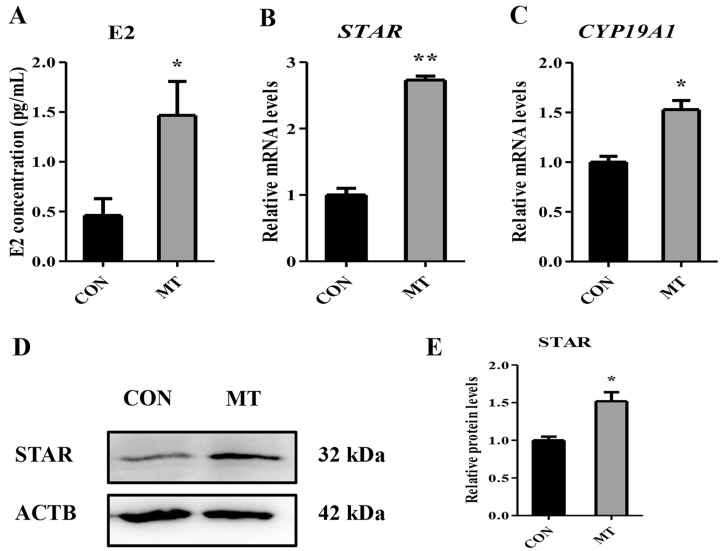
Effects of melatonin on steroidogenesis in granulosa cells. CON group: Untreated. MT group: Cells were treated with 10^−8^ M MT in serum-free medium for 48 h. (**A**) Levels of E2 in the culture medium (n = 6). (**B**,**C**) The mRNA expression levels of *STAR* and *CYP19A1* were determined by qRT-PCR (n = 3). (**D**,**E**) The protein expression levels of STAR were determined by the Western blot analysis (n = 3). Results are expressed as Mean ± SEM. * *p* < 0.05; ** *p* < 0.01.

In the transcriptome data, steroidogenesis acute regulatory protein (STAR) gene was significantly upregulated in the MT group. STAR is responsible for the transport of cholesterol, the prerequisite for steroid hormone synthesis, from the mitochondrial outer membrane to the mitochondrial inner membrane, which is the rate-limiting step of steroid hormone synthesis [35]. With the aim to verify the promoting roles of MT on steroidogenesis of GCs and LCs, the supernatant of GC or CL culture medium was collected and concentrations of E2 and P4 was detected. It was found that MT significantly promoted the secretion levels of E2 in GCs (Figure 10A). qRT-PCR analysis revealed that the mRNA expression levels of *STAR* and *CYP19A1* were significantly upregulated in the MT group compared to the CON group (Figure 10B,C). Additionally, the Western blot assay revealed that the MT group exhibited a higher protein expression level of STAR than the CON group (Figure 10D,E). In LCs, MT markedly improved the secretion levels of P4 (Figure S5A). The expression of *STAR*, *CYP11A1*, and *HSD3B1* were elevated in the MT group compared to the CON group (Figure S5B–E). Overall, the above findings proved that MT could improve steroidogenic competence of GCs and LCs in vitro.

## 4. Discussion

In classic P4-eCG-induced estrus synchronization, fertility is usually lower in anestrous ewes compared to cycling ewes [36]. In the current work, we hypothesized that a 5-day MT implant pretreatment before the vaginal device insertion could improve the reproductive performance of ewes by estrus synchronization of the P4-eCG regime during seasonal anestrus. The findings of the current study offer some evidence in support of our hypothesis.

MT is an indolamine hormone secreted by the pineal gland, which has a powerful antioxidant effect and plays a critical role in modulating the reproductive physiology of animals [18,37]. As expected, we found that the implantation of MT significantly increased the concentration of MT in the serum, indicating that the implantation of MT is effective. We observed that MT implantation altered the levels of several factors in the blood of ewes. MT increased the serum levels of T-AOC, SOD, CAT, GSH, and GSH-PX, and lowered the serum MDA levels in ewes at the time of sponge withdrawal, suggesting that MT implantation 5 days before applying the P4-eCG protocol was enough to increase the levels of serum antioxidant enzymes.

Surprisingly, neither the number of small, medium, large, and total number of follicles nor the diameter of the largest and the second-largest follicles was affected by MT treatment. During the non-breeding season, MT implantation 42 days before applying the P4-eCG protocol in goats tended to increase the number of small and medium follicles as well as the total number of follicles at sponge removal [38]. However, such a role of MT on follicle numbers was not observed in sheep [29]. The effects of MT on follicular dynamics may also be affected by factors such as species and MT implantation time and dose. One limitation of our study is that we did not monitor the final growth of the ovulatory follicle. After sponge withdrawal, MT may have a positive impact on the development of ovulatory follicles before ovulation. This is worthy of further investigation.

The implantation of MT reduced the apoptosis of ovarian cells, which indicates that the implantation of MT has an anti-apoptotic effect on the ovary. Interestingly, in the transcriptomic data, the upregulated DEGs were significantly enriched in BP terms related to DNA damage repair, which can trigger cellular apoptosis. Conversely, the downregulated DEGs were predominantly associated with the natural killer cell-mediated cytotoxicity pathway, which is also implicated in apoptosis regulation. Previous studies have shown that MT reduced ovarian apoptosis in mice [39,40]. These results, together with ours, support the idea that MT has an anti-apoptotic effect in the ovary.

MT implantation increased the CL diameter and serum P4 concentration 8 days after estrus, suggesting that MT can promote CL development and enhance CL P4 synthesis and secretion. These results align with a previous study showing that MT implantation 28 days before the Ovsynch protocol in buffaloes increased the CL diameter and the concentration of P4 in plasma 12 days after AI [41]. Furthermore, a separate investigation demonstrated that MT administration 7 days before superovulation treatment significantly enhanced both the CL diameter and the circulating P4 concentration in goats at 7 days post-ovulation [42]. In sheep, MT implantation 50 days before a synchronized mating enhanced P4 plasma levels on Day 21 of pregnancy [43]. Our results demonstrate that a 5-day MT priming before initiating the conventional P4-eCG protocol is sufficient to promote post-ovulatory CL development. A previous study demonstrated that MT implantation in ewes increased serum IGF-1 concentration after 14 days [28]. In the present study, implantation of MT increased the concentration of IGF-1 in the serum 8 days after estrus. It was shown that IGF-1 can stimulate the CL to secrete P4 [44] and promote luteal development [45,46,47]. Collectively, MT probably promotes luteal development through IGF-1.

The 95% estrus rate in both the CON and MT groups indicated that the conventional P4-eCG protocol alone can induce estrus in sheep outside the natural breeding season, and that a high estrus rate can be achieved through this, which aligns with the findings of previous studies [3,4,5,48,49,50,51]. In the present study, the application of AI following estrus detection led to increased pregnancy and lambing rates in the MT group compared to those in the CON group, although this increase was not significant statistically due to the small animal sample size. This result, to some extent, supported our hypothesis that a 5-day MT implant pretreatment might be sufficient for the improvement of P4-eCG regimen in sheep during seasonal anestrus. Building on these promising preliminary findings, we validated the protocol’s efficacy in a population-scale study on Hu sheep, a breed on which TAI has been established. We introduced MT implantation into the estrus synchronization regimen for this breed. The field trial demonstrated superior reproductive outcomes with MT supplementation, achieving 17% greater pregnancy rates (*p* < 0.05) and 16% higher lambing yields (*p* < 0.05) compared to standard P4-eCG synchronization regimens. Previous studies have demonstrated that MT implantation improved the reproductive performance of ewes [10,28]. However, in those studies, MT implantation was performed for at least 26 days before initiating the conventional P4-eCG protocol. The ewes were naturally mated after estrus, and TAI was not evaluated [10,28]. In the current study, we reduced the interval between MT implantation and initiation of the P4-eCG program to 5 days, and demonstrated its efficacy when used in the TAI regimen.

The reasons for the increase in the reproductive performance may be due to a number of factors. Firstly, MT can promote the maturation of oocytes and the development of early embryos [52,53,54,55,56]. ROS inhibits the oocyte development, leading to oocyte arrest and an increased prevalence of denuded oocytes [57]. Additionally, ROS also suppresses early embryonic development [57]. In this study, MT increased the expression of antioxidant enzymes in serum. It may protect oocytes and embryonic development by scavenging excessive ROS through upregulation of these antioxidant enzymes. Secondly, MT can promote CL to secret P4 [58,59]. P4 is essential for the establishment and maintenance of pregnancy [60,61]. In seasonal breeding of small ruminants, luteal insufficiency manifests predominantly during the period of reproductive quiescence [9]. Suboptimal luteal-phase P4 secretion, a principal contributor to ovine reproductive wastage, precipitates early embryonic mortality in 30–40% of cases due to inadequate endometrial support during critical implantation windows [60,61,62]. In this study, MT implantation greatly increased the CL diameter and serum P4 concentration 8 days after estrus, which may at least partly contribute to the increase in reproductive performance. Finally, MT can also promote embryo implantation and the development of the uterus, placenta, and fetus [15,19,37,43,63,64].

GCs, as the most important cell type in follicles, play a vital role in follicular growth and atresia [65]. The proliferation and apoptosis of GCs are essential for follicular and oocyte development, ovulation, and luteinization, playing a crucial role in regulating oogenesis, ovulation, and female fertility [66]. The proliferation and apoptosis of LCs are closely related to luteal function and regression [67]. Previous studies have found that MT can promote the proliferation of GCs in sheep [68,69,70] and can increase the cell viability of LCs in sows [71,72]. In this study, MT significantly promoted the proliferation of in vitro-cultured GCs and LCs. These findings indicate that MT can promote GC and LC proliferation. It was reported that MT inhibited apoptosis of GCs in ewes [68,69,73]. Furthermore, a previous study has shown that MT suppresses apoptosis of luteinized GCs in mice [74]. In the present study, MT reduced GC and LC apoptosis. Therefore, MT can protect GCs and LCs from apoptosis.

Supraphysiological ROS concentrations can cause oxidative damage, which leads to a decrease in MMP and further results in apoptosis [75]. ROS act as a key inducer of follicular atresia initiation by triggering granulosa cell apoptosis [57]. It was reported that ROS inhibit P4 production in LCs [76]. ROS-triggered apoptotic signaling serves as a pivotal determinant of CL regression kinetics [77]. As a potent antioxidant, MT can directly eliminate ROS or eliminate ROS by stimulating the cellular antioxidant defense system [78]. In this study, MT reduced the ROS levels in GCs and LCs and increased the expression of antioxidant-related genes, indicating that MT can protect GCs and LCs from oxidative stress. In addition, in the transcriptome data, the antioxidant gene *GSTM1* was found to be upregulated in the MT group. As an upstream regulator of GSTM1, NRF2 can promote the expression of GSTM1 and bind to AREs to enhance the expression of antioxidant genes [31,32]. Numerous studies have documented that MT could trigger the activation of the NRF2 signaling cascade [31,68,79,80]. In the present study, we observed that MT activated the NRF2 signaling pathway in GCs and LCs, suggesting that MT may promote the expression of antioxidant-related genes to scavenge ROS through the NRF2 signaling pathway.

In the transcriptomic results, MT upregulated the expression of *STAR*. STAR is responsible for the transport of cholesterol, the prerequisite for steroid hormone synthesis, from the mitochondrial outer membrane to the mitochondrial inner membrane, which is the rate-limiting step of steroid hormone synthesis [35]. In this study, MT promoted STAR expression and E2 secretion in GCs. Preovulatory E2 exerts multifaceted effects on reproductive processes, including follicular development, oocyte maturation, sperm transport dynamics, uterine receptivity, and embryonic viability [81]. Increased E2 secretion during the follicular phase triggers a positive feedback effect, inducing the preovulatory GnRH surge [82,83]. This in turn stimulates the preovulatory LH surge, ultimately leading to ovulation [82,83]. A previous study demonstrated that elevated preovulatory E2 concentrations positively influence pregnancy rates in postpartum cows [84]. Notably, the estrogen signaling pathway was significantly enriched among the upregulated DEGs. This suggests that MT may potentially enhance preovulatory follicular E2 secretion, but it requires to be further determined. In this study, we observed that MT also promoted the expression of STAR in LCs and enhanced the secretion of P4. This finding coincides with our observation that MT promoted luteal development on Day 8 post-estrus. Therefore, MT may enhance the steroid hormone synthesis ability of GCs and LCs by promoting the expression of STAR. In our transcriptome data, the cAMP signaling pathway is enriched in the upregulated DEGs. cAMP/PKA signaling constitutes the principal regulatory axis for steroidogenesis [85]. Future investigations should delineate the spatiotemporal dynamics of MT-mediated cAMP/PKA modulation in ovine GCs and LCs.

## 5. Conclusions

In conclusion, MT implantation 5 days before the start of conventional P4-eCG protocol can improve the reproductive performance of sheep during seasonal anestrus. Furthermore, MT implantation has great impact on the ovaries, such as altering the gene expression of ovarian cells and promoting the proliferation of GCs and LCs while inhibiting their apoptosis. Probably through the activation of the NRF2 signaling pathway, MT enhances the antioxidant capacity of GCs and LCs and improves their steroid hormone synthesis ability. These findings offer novel evidence supporting the functional role of MT in mammalian reproduction and would be informative for optimizing the estrus synchronization in sheep.

## Figures and Tables

**Table 1:** Effects of melatonin implantation on reproductive traits of Mongolian sheep (experiment 1).

Parameters	CON Group (n = 20)	MT Group (n = 20)	*p* Value
Estrus rate (%)	95.00 (19/20)	95.00 (19/20)	n.s.
Ovulation rate (n)	1.00 ± 0.00	1.14 ± 0.14	n.s.
Pregnancy rate (%)	56.25 (9/16)	86.00 (13/15)	n.s.
Lambing rate (%)	56.25 (9/16)	86.00 (13/15)	n.s.
Litter size (n)	1.00 ± 0.00 (9/9)	1.00 ± 0.00 (13/13)	n.s.

CON group: FGA (45 mg) sponge for 14 days + 330 IU eCG at sponge withdrawal. MT group: following 5 days of MT implantation, the same protocol as the CON group was applied. Note that 3 and 4 estrus ewes underwent surgical removal of ovaries in the CON and MT groups, respectively, and thus these ewes were not inseminated. Estrus rate: number of estrous ewes/number of total ewes. Ovulation rate: number of CL/number of ovulated ewes. Pregnancy rate: number of pregnant ewes/number of inseminated estrous ewes. Lambing rate: number of lambed ewes/number of inseminated estrous ewes. Litter size: number of total lambs/number of lambed ewes. n.s.: not significant (*p* > 0.05).

**Table 2:** Effects of melatonin implantation on reproductive traits of Hu sheep (experiment 2).

Parameters	CON Group (n = 85)	MT Group (n = 85)	*p* Value
Pregnancy rate (%)	50.59 ^b^ (43/85)	68.23 ^a^ (58/85)	*p* = 0.019
Lambing rate (%)	47.06 ^b^ (40/85)	63.53 ^a^ (54/85)	*p* = 0.031

CON group: FGA (45 mg) sponge for 14 days + 330 IU eCG at sponge withdrawal. MT group: Following 5 days of MT implantation, the same protocol as the CON group was applied. Pregnancy rate: number of pregnant ewes/number of total ewes. Lambing rate: number of lambed ewes/number of total ewes. Within a row, values with different superscript lowercase letters differ significantly (*p* < 0.05).

## Data Availability

The datasets generated and analyzed during the current study can be obtained from the corresponding author upon reasonable request.

## Supplementary Materials

The following supporting information can be downloaded at https://www.mdpi.com/article/10.3390/antiox14070895/s1.
