# Supplementary material for: Melatonin Implantation Improves the Reproductive Performance of Estrus-Synchronized Ewes During Seasonal Anestrus and Enhances the Antioxidant and Steroidogenic Capacities of Granulosa and Luteal Cells
Source: Antioxidants (Basel). 2025 Jul 21;14(7):895. doi: 10.3390/antiox14070895 (PMC12292622; doi:10.3390/antiox14070895)
Supplement: Supplementary file 1 [file antioxidants-14-00895-s001.zip › antioxidants-3699407-supplementary.pdf]

# Supplementary materials

# Melatonin Implantation Improves the Reproductive Performance of Estrus-Synchronized Ewes During Seasonal Anestrus and Enhances the Antioxidant and Steroidogenic Capacities of Granulosa and Luteal Cells

**Zengyi Duan, Menghao Liu, Junjin Li, Kexiong Liu, Qi Qi, Zhixuan Yu, Hadia Akber Samoo, Chunxin Wang and Jian Hou ***

State Key Laboratory of Animal Biotech Breeding, College of Biological Sciences, China Agricultural University, Yuan-Ming-Yuan West Road, Haidian District, Beijing 100193, China; zengyiduan@163.com (Z.D.); liumenghao@cau.edu.cn (M.L.); liyuhailan@126.com (J.L.); liukexiong2023@163.com (K.L.); qiqi2017@cau.edu.cn (Q.Q.); yuzhixuan@cau.edu.cn (Z.Y.); hadiaakbersamoo@stu.zafu.edu.cn ( H.A.S.); wcxjlsnky@163.com (C.W.).

*Correspondence: houjian@cau.edu.cn; Tel.:+86-10-62733355

**Table S1.** Details of the primer sequences used in this research.

| Genes | Sequence Primers (5′–3′) | Size (bp) |
|---|---|---|
| *PCNA* | Forward:TGCAGATGTACCCCTTGTTGT<br>Reverse:AGACTTAAGTGTGTGCTGGC | 173 |
| *CDK4* | Forward:TGCTGGGATGCTGACTTTTAACCC<br>Reverse:CTCTGCGTCACCTTCTGCCTTG | 95 |
| *CDK6* | Forward:CTTCGGCCTTGCTCGCATCTAC<br>Reverse:AATATGCAGCCAACGCTCCAGAG | 138 |
| *CCND1* | Forward:CTCGGTGTCCTACTTCAAGTGTGTG<br>Reverse:TCGCAGACCTCCAGCATCCAG | 87 |
| *CDKN1A* | Forward:ACGTCTCAGGAGGACCACTT<br>Reverse:TGGTAGAAATCTGTCATGCTGGT | 143 |
| *CDKN1B* | Forward:TACTTGGGTCTCAGGCAAAC<br>Reverse:GCTCTTTTGTTTTGAGGAGAGG | 148 |
| *CAT* | Forward:ACAATGTCACTCAGGTGCGG<br>Reverse:CTGTGCGTCTTTCAGATGGC | 101 |
| *GPX1* | Forward:GTTTGGGCATCAGGAAAACGC<br>Reverse:GTCATGAGAGCAGTGGCGTC | 195 |
| *GPX4* | Forward:GCCGAGTGTGGTTTACGGAT<br>Reverse:TCCATTTGATGGCGTTTCCC | 220 |
| *SOD1* | Forward: AAGGGAGATAAAGTCGTCGTA<br>Reverse: TTCACATTGCCCAGGTCTC | 191 |
| *SOD2* | Forward: CCGTCAGCCTTACACCAAGT<br>Reverse: CAAGCCACGCTCAGAAACAC | 112 |
| *NRF2* | Forward:TGTGGAGGAGTTCAACGAGC<br>Reverse:CGCCGCCATCTTGTTCTTG | 103 |
| *NQO1* | Forward:TTCTGTGGCTTCCAGGTCTT<br>Reverse:TCCAGACGTTTCTTCCATCC | 102 |
| *HO-1* | Forward:GGCAGCAAGGCACAAGACTC<br>Reverse:GCACACTCGCATTCACATGG | 159 |
| *CASP3* | Forward:AACGTTGTGGCTGAACGTAAA<br>Reverse:AGCTCGTGAAGGTTTCCCTG | 274 |
| *BAX* | Forward:GCCCTTTTGCTTCAGGGTTTC<br>Reverse:TCAGACACTCGCTCAGCTTC | 121 |

| | | |
|---|---|---|
| *BCL2* | Forward:CAGGAGAAATCAAACAGGG<br>Reverse:GTGTGTGGAGAGCGTCAAC | 171 |
| *FASLG* | Forward:CCACGTGGCTGGTATCAACT<br>Reverse:GGCTGACAGCAAAACAGGTG | 108 |
| *CASP8* | Forward:TGAAGGTTCCAGGATTCGCC<br>Reverse:GGCTTAGGAACTTGAGGGCA | 136 |
| *JUN* | Forward:GCTTCCAAGTGCCGGAAAAG<br>Reverse:GCTGCGTTAGCATGAGTTGG | 184 |
| *FOS* | Forward:GGGAGGACCTTATCTGTGCG<br>Reverse:GGGAGGACCTTATCTGTGCG | 125 |
| *GSTM1* | Forward:GACTTCATTACCCGTTTTGAGACC<br>Reverse:CCCACACGGCAAGCTTTAGA | 100 |
| *KCNK3* | Forward:GGAGATGATCGAGCGGCAG<br>Reverse:CGTGGCCGTAGCCGATG | 197 |
| *PARP3* | Forward:AGTCGGCTGGCTATGTTACT<br>Reverse:ATCTAGCTCCAGCTCGGTGT | 203 |
| *ADCYAP1* | Forward:GGAGATACCTGCAGACGCTC<br>Reverse:TGTCAGTGAAGATGCCGTCC | 114 |
| *CYP11A1* | Forward:CGCTTTGCCTTTGAGTCCATC<br>Reverse:TGAGCAGAGGGACACTGGTA | 136 |
| *HSD3B1* | Forward:GGAGACATTCTGGATGAGCAG<br>Reverse:TCTATGGTGCTGGTGTGGA | 200 |
| *CYP19A1* | Forward:GGTCATCCTGGTCACCCTTCTG<br>Reverse:GCCGGTCGCTGGTCTCGTCTGG | 119 |
| *STAR* | Forward:AGCATTGACCTCAAGGGATGG<br>Reverse:GCCTTCAACACCTGGCTTCA | 133 |
| *ACTB* | Forward:GCGGCATTCACGAAACTACC<br>Reverse:GGGGCGCGATGATCTTGA | 183 |

**Table S2.** Details of antibodies used in the present study.

| Antibody | Company | Cat No. | Host | Dilution |
|---|---|---|---|---|
| ACTB | QuaYad | QYA10733A | mouse | 1:5000 |
| β-Tubulin | TransGen Biotech | HC101 | mouse | 1:5000 |
| GSTM1 | Sangon Biotech | D126782 | rabbit | 1:1000 |
| STAR | Proteintech | 12225-1-AP | rabbit | 1:1000 |
| HSD3B1 | Santa Cruz | SC-515120 | mouse | 1:1000 |
| NRF2 | Proteintech | 16396-1-AP | Rabbit | 1:1000 |
| NQO1 | Sangon Biotech | D161049 | rabbit | 1:1000 |
| HO-1 | Abclonal | A1346 | rabbit | 1:1000 |
| Goat Anti-Rabbit IgG | Sangon Biotech | D110058 | Goat | 1:5000 |
| Goat Anti-Mous IgG | Sangon Biotech | D110103 | Goat | 1:5000 |

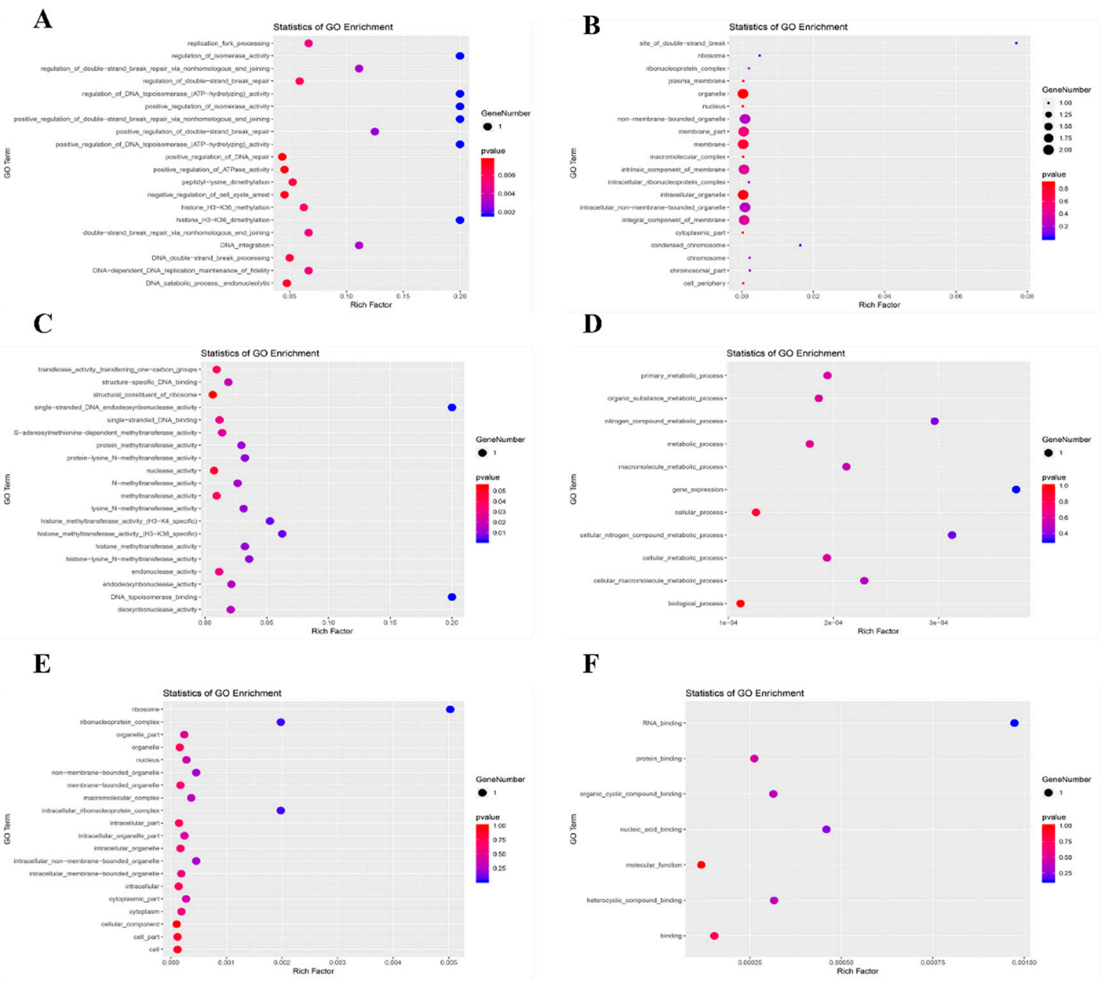


**Figure S1.** Gene Ontology (GO) enrichment analysis of differentially expressed genes (DEGs). (A) GO enrichment analysis of upregulated DCGs in the GO term of biological process (BP). (B) GO enrichment analysis of upregulated DCGs in the GO term of cellular component (CC). (C) GO enrichment analysis of upregulated DCGs in the GO term of molecular function (MF). (D) GO enrichment analysis of downregulated DCGs in the GO term of BP (E) GO enrichment analysis of downregulated DCGs in the GO term of CC. (F) GO enrichment analysis of downregulated DCGs in the GO term of MF. The 20 most significantly enriched terms are showed. The vertical coordinates and horizontal coordinates indicate GO term and enrichment factor, respectively. The size of the circle indicates the number of enriched DEGs. The color of the circle represents the *P*-value; the darker the color, the smaller the *P*-value.

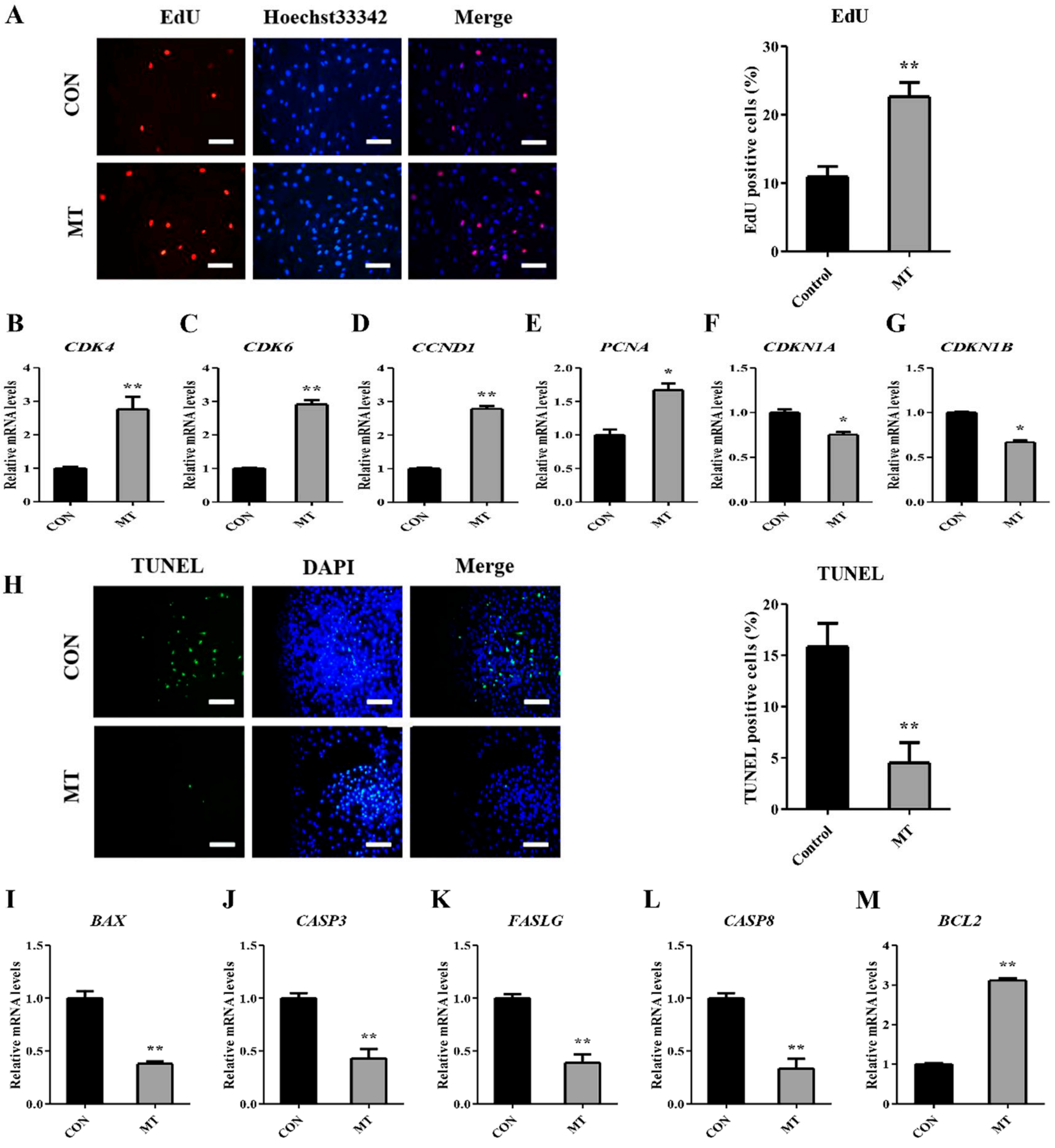


**Figure S2**. Effect of melatonin on cell proliferation and apoptosis of luteal cells. CON group: Untreated. MT group: Cells were treated with $10^{-8}$ M MT in serum-free medium for 48 h. (A) Representative images and quantified results of EdU. Scale bars = 50 μm. (B-G) The mRNA expression levels of *CDK4*, *CDK6*, *CCND1*, *PCNA*, *CDKN1A*, and *CDKN1B* were determined by qRT-PCR. (H) Representative images and quantified results of TUNEL. Scale bars = 50 μm. (I-M) The mRNA expression levels of *BAX*, *CASP3*, *FASLG*, *CASP8*, and *BCL2* were determined by qRT-PCR. Results are expressed as Mean ± SEM (n = 3). * $P < 0.05$; ** $P < 0.01$.

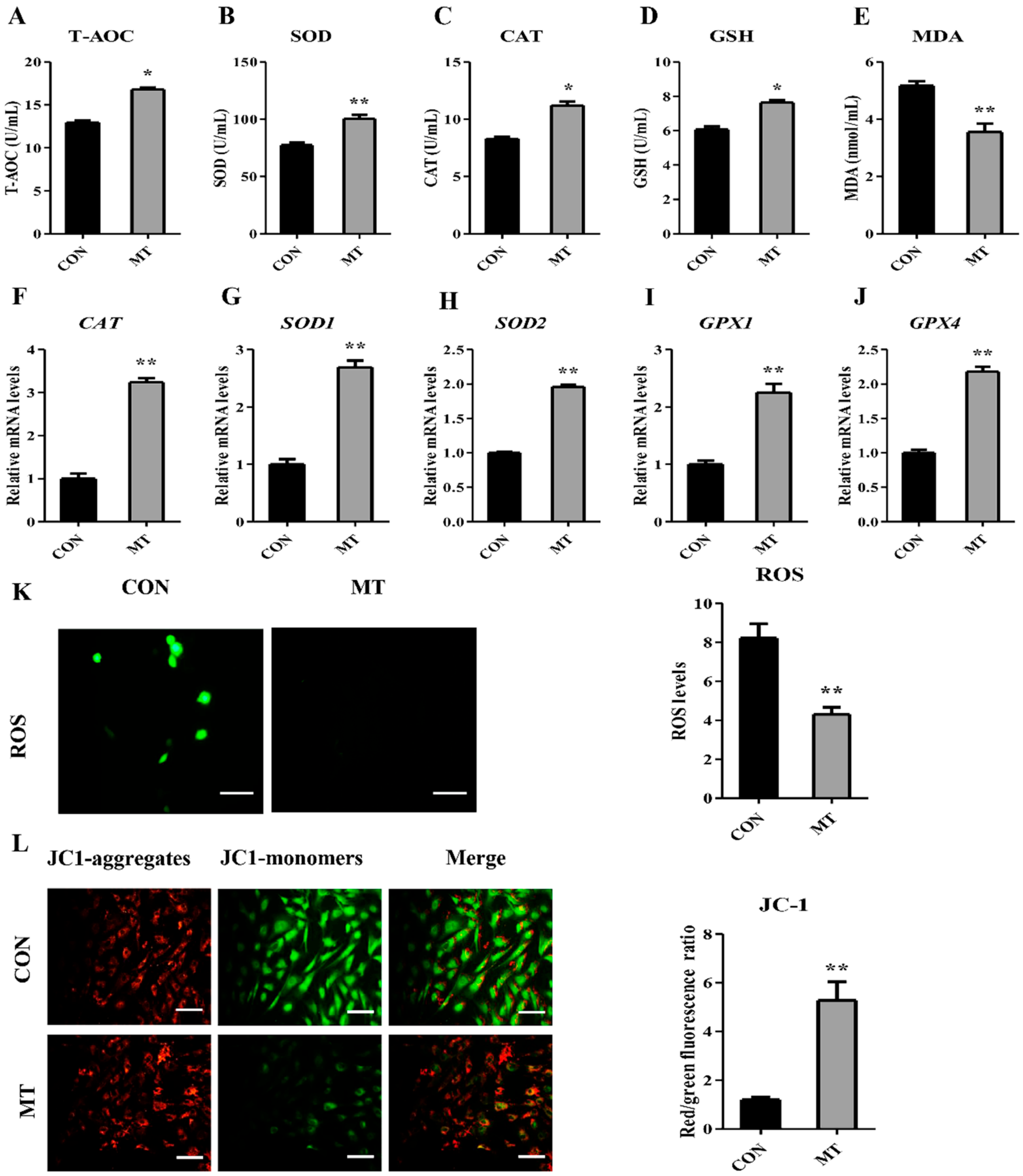


**Figure S3.** Effect of melatonin on the antioxidant capacity of luteal cells. CON group: Untreated. MT group: Cells were treated with $10^{-8}$ M MT in serum-free medium for 48 h. (A-E) Levels of T-AOC, SOD, CAT, GSH, and MDA in the culture medium (n = 6). (F-J) The mRNA expression levels of *CAT*, *SOD1*, *SOD2*, *GPX1*, and *GPX4* were determined by qRT-PCR (n = 3). (K) Representative images and quantified results of ROS (n = 3). Scale bars = 50 μm. (L) Representative images and quantified results of MMP (n = 3). Scale bars = 50 μm. Results are expressed as Mean ± SEM. * $P < 0.05$; ** $P < 0.01$.

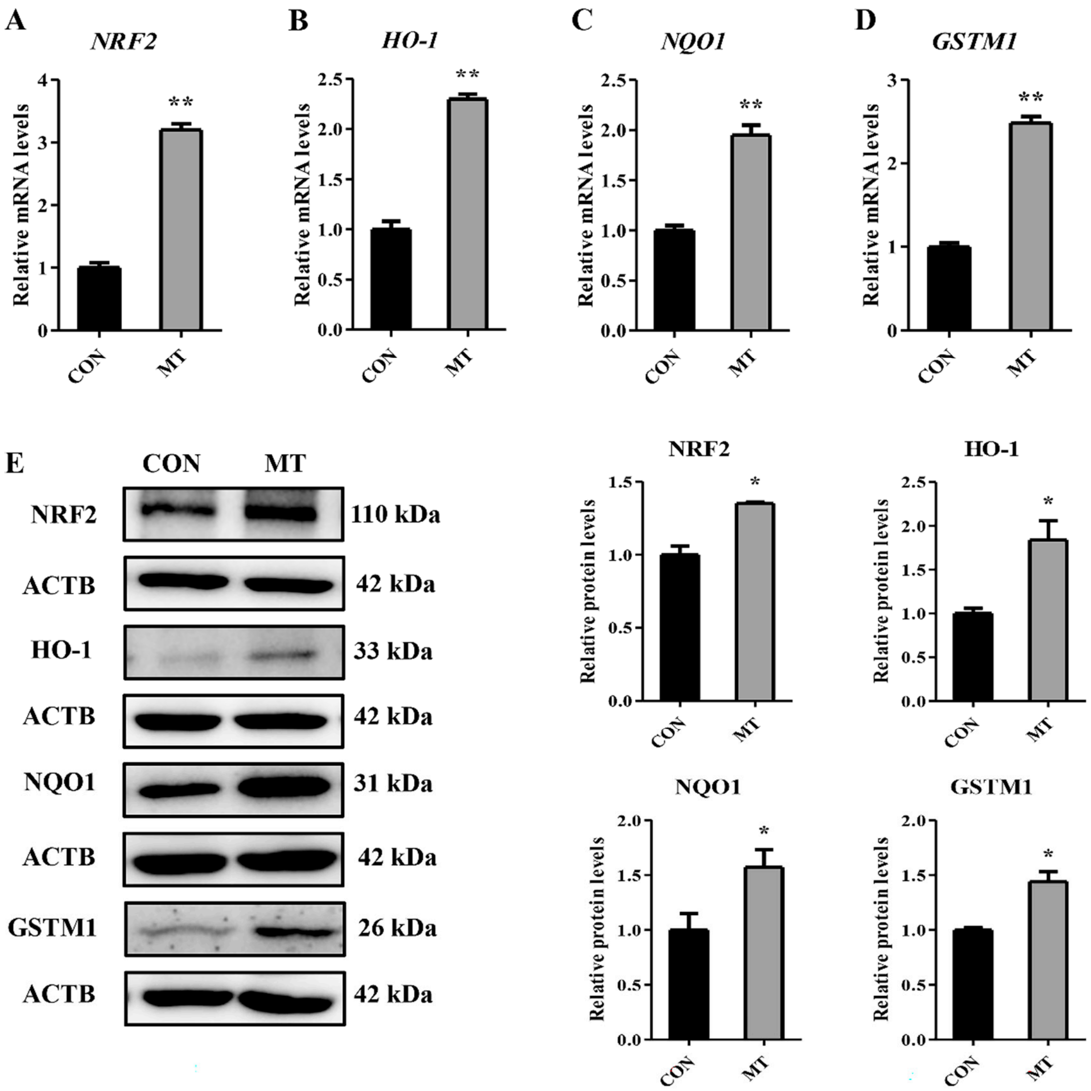


**Figure S4.** Effect of melatonin on NRF2 signaling pathway in luteal cells. CON group: Untreated. MT group: Cells were treated with $10^{-8}$ M MT in serum-free medium for 48 h. (A-D) The mRNA expression levels of *NRF2*, *HO-1*, *NQO1*, and *GSTM1* were determined by qRT-PCR. (E) The protein expression levels of NRF2, HO-1, NQO1, and GSTM1 were determined by the Western blot analysis. Results are expressed as Mean ± SEM (n = 3). * $P < 0.05$; ** $P < 0.01$.

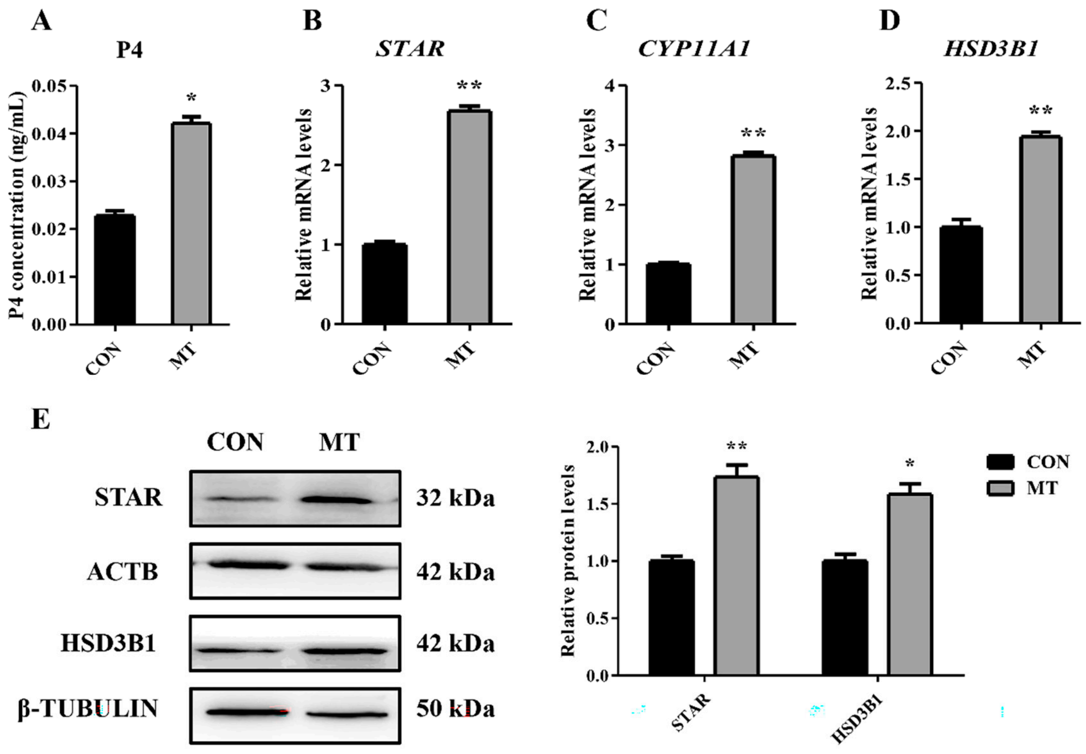


**Figure S5.** Effect of melatonin on steroidogenesis in luteal cells. CON group: Untreated. MT group: Cells were treated with $10^{-8}$ M MT in serum-free medium for 48 h (A) Levels of P4 in the culture medium (n = 6). (B-D) The mRNA expression levels of *STAR*, *CYP11A1*, and *HSD3B1* were determined by qRT-PCR (n = 3). (D-E) The protein expression levels of STAR and HSD3B1 were determined by the Western blot analysis (n = 3). Results are expressed as Mean ± SEM. * $P < 0.05$; ** $P < 0.01$.
